# Efficacy and Safety of Apatinib in Advanced Hepatocellular Carcinoma: A Multicenter Real World Retrospective Study

**DOI:** 10.3389/fphar.2022.894016

**Published:** 2022-05-17

**Authors:** Zhuangzhuang Zheng, Zijing Liu, Haifeng Zhang, Xiao Guo, Xiaojing Jia, Jianfeng Wang, Lingbin Meng, Ying Xin, Xin Jiang

**Affiliations:** ^1^ Department of Radiation Oncology, The First Hospital of Jilin University, Changchun, China; ^2^ Jilin Provincial Key Laboratory of Radiation Oncology and Therapy, Changchun, China; ^3^ NHC Key Laboratory of Radiobiology, School of Public Health of Jilin University, Changchun, China; ^4^ Department of Interventional Therapy, The First Hospital of Jilin University, Changchun, China; ^5^ Department of Radiation Oncology, Jilin Cancer Hospital, Changchun, China; ^6^ Department of Radiation Oncology, The Second Hospital of Jilin University, Changchun, China; ^7^ Department of Radiation Oncology, China-Japan Union Hospital of Jilin University, Changchun, China; ^8^ Department of Hematology and Medical Oncology, Moffitt Cancer Center, Tampa, FL, United States; ^9^ Key Laboratory of Pathobiology, Ministry of Education, Jilin University, Changchun, China

**Keywords:** apatinib, hepatocellular carcinoma, vascular endothelial growth factor-2 (VEGFR-2), transcatheter arterial chemoembolization (TACE), progression-free survival, overall survival, targeted therapy

## Abstract

**Background and Purpose:** Apatinib is a novel antiangiogenic agent that can target vascular endothelial cell growth factor 2. The aim of our study was to evaluate the efficacy and safety of apatinib mesylate in the treatment of advanced hepatocellular carcinoma (HCC) in the real world.

**Methods:** We retrospectively analyzed 178 patients with advanced HCC who had been treated with apatinib mesylate from January 2017 to March 2020. The primary outcome indexes were progression-free survival (PFS) and overall survival (OS), and the secondary outcome indexes were overall response rate (ORR), disease control rate (DCR), and incidence of treatment-related adverse reactions.

**Results:** Univariate analysis showed that patients with third-line treatment (*p* <0.001), alpha fetoprotein (AFP) ≥400 ng/ml (*p* <0.05), distant metastasis (*p* <0.05), portal vein tumor thrombus (PVTT) (*p* <0.05), and apatinib monotherapy (*p* <0.001) had shorter survival. Multivariate analysis confirmed that third-line drugs, PVTT, and combination therapy were independent prognostic factors for PFS in all patients. Univariate analysis showed that Eastern Cooperative Oncology Group (ECOG) scores (*p* <0.05), line of apatinib (*p* <0.001), AFP (*p* <0.001), tumor progression (*p* <0.05), PVTT (*p* <0.05), and combination therapy (*p* <0.001) may impact the OS. Multivariate analysis proved that AFP, PVTT, and combination therapy were independent prognostic factors for OS. The most common adverse reactions were secondary hypertension (29.21%), symptoms of fatigue (16.85%), hand and foot syndrome (16.29%), vomiting (14.04%), liver dysfunction (6.18%), and proteinuria (6.74%). Most of the adverse reactions were Grade 1 or 2.

**Conclusion:** Apatinib mesylate is an effective treatment for advanced HCC, and its adverse reactions are relatively mild. Line of apatinib, PVTT, AFP level, and combination therapy were independent prognostic factors for patients with advanced HCC who were treated with apatinib.

## Introduction

As one of the most common malignancies with a poor prognosis, the incidence of liver cancer continues to increase (Siegel et al., 2021). Hepatocellular carcinoma (HCC) is a major type of primary liver cancer. HCC has become an important problem affecting human health and quality of life worldwide. In China, the hepatitis B virus (HBV) is a major risk factor for primary HCC; more than 80% of primary HCC patients are HBV hepatitis B surface antigen (HBsAg)‐positive (Kim et al., 2020). Besides HBV, high alpha fetoprotein (AFP), portal vein tumor thrombus (PVTT), and hepatitis C virus (HCV) also affect the prognosis of patients (Bruix et al., 2017a; Bruix et al., 20172018; Zheng et al., 2020a; Fu et al., 2020). Surgery is the preferred treatment for early primary liver cancer. Image-guided ablation, radiofrequency ablation, and microwave ablation are also applicated in the treatment of early HCC and the alternative treatment of surgery (Llovet et al., 2021). However, despite the best efforts to remove the tumor surgically, tumor recurrence still occurs in more than 50% of patients within 5 years of surgery (Heimbach et al., 2018). Outcomes of liver transplantation were considered superior than resection. However, organ shortage with prolonged waiting times plagued HCC patients, leading to tumour progression (Franssen et al., 2014). Radiotherapy can be used for HCC patients with different sizes and stages, particularly with small tumours not amenable to resection or transplantation. But radiotherapy only applied to selected patients (Wahl et al., 2016; Yang et al., 2019a). More research was needed to determine the best radiation modality and combination treatment options. Additionally, due to the insidious onset and insignificant early symptoms of HCC, most patients are diagnosed with advanced HCC. Transcatheter arterial chemoembolization (TACE) is mainly used in patients with unresectable HCC, and could effectively inhibit tumor progression (Burrel et al., 2012; Lencioni et al., 2016). However, TACE induces hypoxia in HCC tissues and increases the level of the pro-angiogenic factor, vascular endothelial growth factor (VEGF), in the remaining HCC tissues, leading to a significant neovascularization response and recurrence after treatment (Bergers et al., 2000). High VEGF receptor (VEGFR) expression promotes tumor nutrient supply, growth, metastasis, and recurrence, suggesting a poor prognosis. The previous monotherapy for first-line standard of care for patients with advanced and recurrent HCC are sorafenib and lenvatinib (Kudo et al., 2018; Benson et al., 2021). VEGF plays an important role in the occurrence and development of HCC; furthermore, drugs targeting VEGF and VEGF receptors have been used in patients with advanced HCC (Du et al., 2017). Attilizumab and antiangiogenic drugs bevacizumab have been recommended as first-line treatments for advanced HCC due to better efficacy ((Pawlik et al., 2004; Li et al., 2013; Nishikawa et al., 2013), (Pawlik et al., 2004; Li et al., 2013; Nishikawa et al., 2013). Sorafenib, a multitargeted tyrosine kinase inhibitor (TKI) and, previously, the only Food and Drug Administration (FDA)-approved, first-line-targeted drug for patients with advanced HCC (Llovet et al., 2008), extended overall survival (OS) by less than 3 months in some clinical trials (19). In the phase III randomized REFLECT trial, lenvatinib, s howed a median OS (mOS) of 13.6 months, compared with 12.3 months in the sorafenib group (Kudo et al., 2018). Based on the results, the FDA approved lenvatinib in 2018 as the first-line treatment of patients with unresectable HCC. Regorafenib, cabozantinib, and ramucirumab are recommended as second-line systemic therapies for HCC patients who have received sorafenib treatment according to the 2021 National Comprehensive Cancer Network® (NCCN) guidelines on hepatobiliary cancers (Benson et al., 2021). In addition, based on the phase Ib/II studies, three additional treatments, namely nivolumab, pembrolizumab and ipilimumab, have been approved by the FDA after first-line treatment with sorafenib (El-Khoueiry et al., 2017; Zhu et al., 2018; Yau et al., 2020). The latest CheckMate 459 study showed that first-line nivolumab treatment (16.4 months) did not significantly improve OS compared with sorafenib (14.7 months) (Yau et al., 2022). Nivolumab might be considered as an alternative therapeutic option for patients in whom TKIs and antiangiogenic drugs are contraindicated or have risks. However, many clinical studies have shown that a considerable number of patients with HCC are not sensitive to sorafenib, and the overall efficacy is far from satisfactory (Zhu et al., 2017; Xu et al., 2020). And current therapies would cause a variety of potential adverse reactions. Therefore, we still need to discover new effective and safe drugs for HCC treatment.

Apatinib, a novel TKI, inhibits the activity of VEGFR-2 tyrosine kinase in a highly selective manner, thereby inhibiting tumor growth by inhibiting tumor angiogenesis. Apatinib can inhibit tumor cell apoptosis and cell proliferation *in vitro* by blocking the VEGFR pathway and by inhibiting the growth of metastatic tumors *in vivo* (Li et al., 2018). Several clinical trials have shown that apatinib is effective in a variety of solid tumors. In China, apatinib is approved as a follow-up treatment for patients with advanced gastric adenocarcinoma or gastroesophageal junction adenocarcinoma that has progressed or relapsed after at least one round of seed system chemotherapy. In stage Ⅱ and Ⅲ studies, apatinib has been shown to be effective and safe in patients with gastric cancer (Zhang et al., 2017; Zheng et al., 2020b). A real world retrospective study by Zhang et al. (Zhang et al., 2017) showed that the efficacy and safety of apatinib were similar to the results of previous clinical trials. Apatinib therapy is beneficial and tolerable in patients with advanced gastric cancer who don’t respond to systemic therapy. In recent years, several studies have demonstrated its efficacy and safety in HCC, especially in patients insensitive to sorafenib (Liao et al., 2019). Apatinib increases radiosensitivity of HCC and decreases tumor growth via the suppression of the radiation-induced PI3K/AKT pathway. Additionally, combination immunization or TACE with apatinib also show good efficacy (Xu et al., 2021). TACE therapy creates an anoxic environment for the tumor, inducing high VEGFR expression and angiogenesis; this was the target attacked by apatinib. Therefore, in clinical treatment, physicians can develop an appropriate treatment combination and treatment dose based on the patient’s condition. Apatinib has been used in the treatment of advanced HCC, but its efficacy and combination therapy have not been systematically evaluated. To evaluate the efficacy and safety of apatinib in the real world, we retrospectively analyzed 178 patients with advanced HCC who had received apatinib from 2017 to 2020, and determined the influencing factors for progression-free survival (PFS) and OS. The results of this study provided a theoretical basis for the application of apatinib in patients with advanced HCC.

## Patients and Methods

### Patients

We screened 185 patients receiving apatinib for unresectable or relapsed HCC from the First Hospital of Jilin University, China-Japan Union Hospital of Jilin University, the Second Hospital of Jilin University, and Jilin Cancer Hospital between January 2017 and August 2020. Finally, the study included 178 eligible patients (Supplementary figure S1) The inclusion criteria were: age ≥18 years; pathological confirmation as HCC; the duration of apatinib treatment ≥1 month; at least one measurable lesion according to Response Evaluation Criteria In Solid Tumors (RECIST) version 1.1; Eastern Cooperative Oncology Group performance status (ECOG) 0–2; and Child–Pugh score ≥ B. Key exclusion criteria were: other malignancies that had been diagnosed before this study; serious respiratory, cardiovascular or kidney disease; pregnant and lactating women. The study was conducted according to Good Clinical Practices and was approved by the ethics committee of the institution.

### Ethics approval and consent to participate

All experimental protocols were approved by the Ethics Committee of the First Hospital of Jilin University. This was a retrospective study; all the patients received a normal standard treatment plan and were followed up after the treatment. No harm was caused to the patients as a result of the study procedures, therefore, the requirement for informed consent was waived.

### Treatment

Apatinib was produced by Jiangsu Hengrui Medicine Co., Ltd (Jiangsu province) as tablets to be administered daily, orally. Patients were treated with apatinib 250 mg or 500 mg daily until disease progression or till it became intolerable. During apatinib treatment, the physicians combined TACE and immunotherapy according to the patients' condition. Body status, blood pressure, complete blood count, and liver and kidney function were monitored during the treatment.

### Efficacy and safety assessments

We collected the clinical and radiological data, such as tumor response, survival time, adverse reactions, combination therapy, at baseline and at 1 month after treatment initiation. Tumor response and adverse reactions were evaluated according to RECIST 1.1 and Common Terminology Criteria for Adverse Events 5.0 (CTCAE 5.0), respectively.

### Data analysis

Statistical analyses were performed using SPSS version 25.0 (IBM Corp., Armonk, N.Y., United States). Categorical variables are expressed as numbers or percentages (%), and continuous variables are expressed as the mean ± standard deviation. Survival analysis was calculated using Kaplan–Meier survival curves on GraphPad Prism 8.0.1 (GraphPad Software, San Diego, California, United States). Univariate and multivariate Cox proportional hazards regression analyses were used to predict the prognostic factors for PFS and OS. A value of *p* < 0.05 was considered statistically significant.

## Results

### Patients characteristics

A total of 178 patients with advanced HCC were included in this retrospective study. Patient characteristics at baseline were summarized in Table 1. Of these, 146 patients (82.02%) were male, and 32 (17.98%) were female. The median age of these patients was 58 years. The dosage of apatinib was determined by the attending physician based on the patient’s medical condition. Of the total, 174 patients (97.75%) were administered a 250 mg dosage and 4 patients (2.25%), the 500 mg dosage. Additionally, 25 patients (14.04%) were treated with apatinib in combination with immunotherapy and 103 patients (57.87%) were treated with TACE at least once during treatment with apatinib.

**TABLE 1:** Baseline characteristics of the patients treated with apatinib.

Characteristics	*n* = 178
Sex	–
Men	146 (82.02%)
Women	32 (17.98%)
Age	–
<58	87 (48.88%)
≥58	91 (51.12%)
Tumor status	–
Intrahepatic metastasis	108 (60.67%)
Distant metastasis	44 (24.72%)
Recurrence	26 (14.61%)
Cancer embolus	–
Present	29 (16.29%)
Absent	149 (83.71%)
Hepatitis (HBV/HCV)	–
Present	156 (87.64%)
Absent	22 (12.36%)
Smoking history	–
Yes	32 (17.98%)
No	146 (82.02%)
Hypertension history	–
Yes	15 (8.43%)
No	163 (91.57%)
Drinking	
Yes	33 (18.54%)
No	145 (81.46%)
AFP (ng/ml)	–
<400	105 (58.99%)
≥400	73 (41.01%)
ECOG score	–
0–1	142 (79.77%)
2	36 (20.23%)
Child-Pugh class	–
A	146 (82.02%)
B	32 (17.98%)
Line of apatinib	–
First-line	56 (31.46%)
Second-line	112 (62.92%)
Third-line	10 (5.62%)
Drug dose	–
250 mg	174 (97.75%)
500 mg	4 (2.25%)
Combination therapy	–
Apatinib	50 (28.09%)
Apatinib + immunotherapy	25 (14.04%)
Apatinib + TACE	103 (57.87%)

### Efficacy of apatinib in the treatment of HCC

**FIGURE 1:**
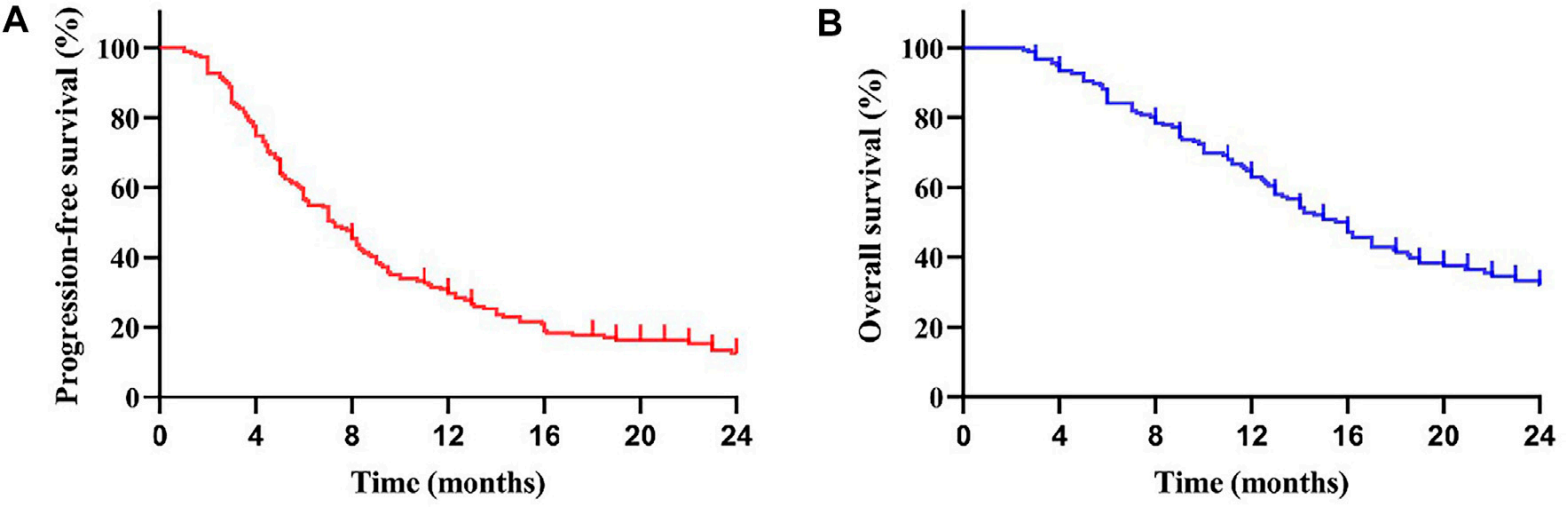
Kaplan-Meier curve for progression-free survival (PFS) **(A)** and overall survival (OS) **(B)** of the patients: The mPFS was 7.0 months (95% CI: 5.69–8.31 months) in all subjects. The mOS was 16.0 months (95% CI: 14.17–17.83 months).

All patients in our efficacy analyses were treated with apatinib for at least one month. A total of 24 months of follow-up was performed. The result in Table 2 shows that complete response (CR) did not occur in any of the patients; 28 patients (15.73%) showed partial response (PR); 103 patients (57.87%) showed stable disease (SD); and 47 patients (26.40%) had progressive disease (PD). The overall response rate (ORR) was 15.73%. The disease control rate (DCR) was 73.60%. In the 28 patients with PR, 27 had 250 mg apatinib as the first/second-line treatment, and 21 patients were treated using combined immunotherapy or TACE. The Kaplan–Meier curves of total PFS (a) and OS (b) are shown in Figure 1. Patients had an overall median PFS (mPFS) of 7.0 months and an overall mOS of 16.0 months.

**TABLE 2:** Efficacy of apatinib in patients (*n* = 178).

Responses	n (%)
Complete response (CR)	0 (0%)
Partial response (PR)	28 (15.73%)
Stable disease (SD)	103 (57.87%)
Progressive disease (PD)	47 (26.40%)
Overall response rate (ORR)	15.73%
Disease control rate (DCR)	73.60%

ORR: CR + PR; DCR: CR + PR + SD.

### Prognostic factors affecting OS and PFS

**FIGURE 2:**
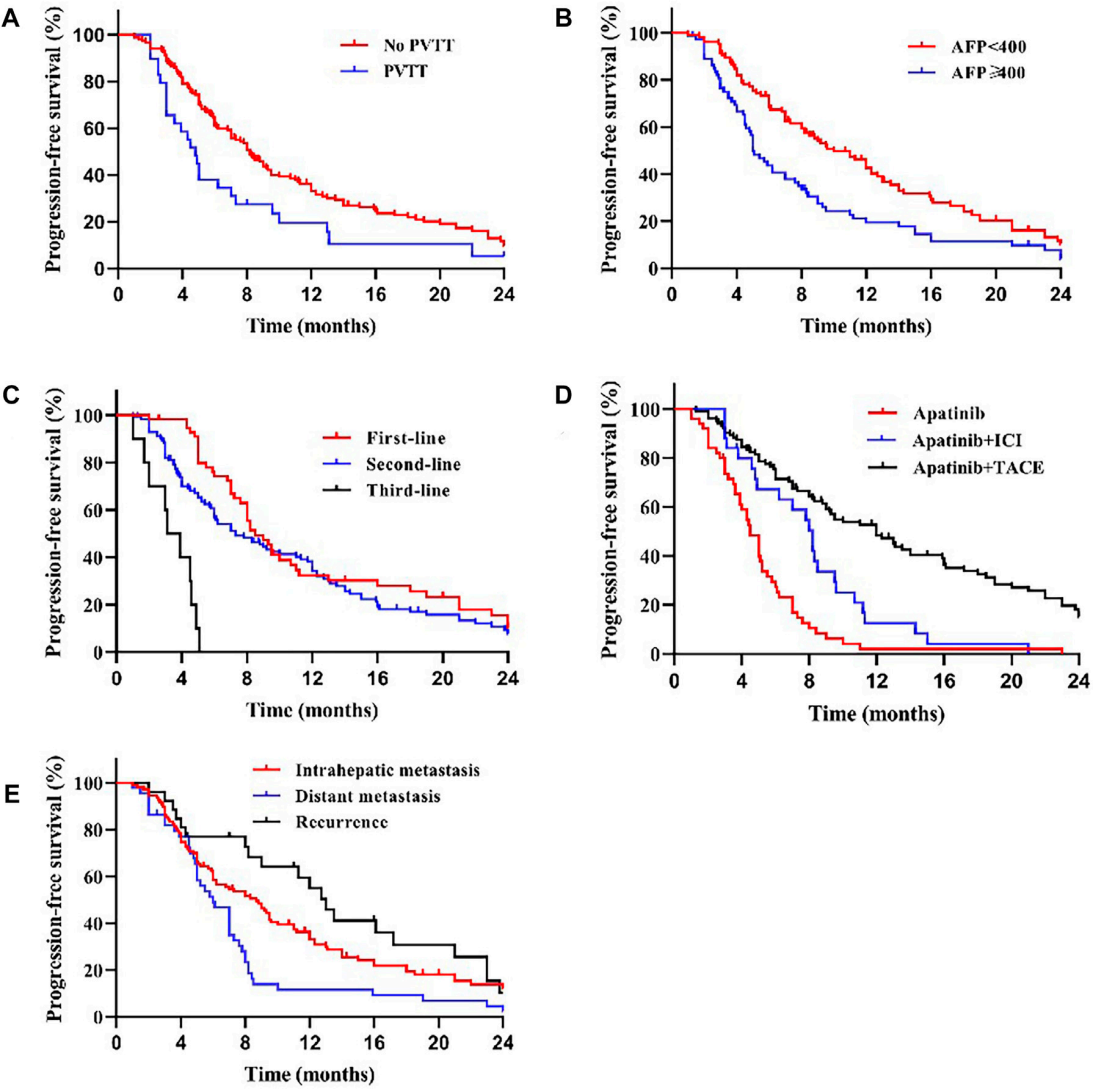
Kaplan–Meier curves showing the PFS of patients by univariate analysis with advanced HCC treated with apatinib, including significantly different results. **(A)** Comparison of PFS between patients with PVTT (present) and without PVTT (absent) before the apatinib therapy. The mPFS was 4.8 months (95% CI: 3.75–5.86) for treatment with PVTT versus 8.0 months (95% CI: 7.06–8.94) for treatment without PVTT (*p* < 0.05). **(B)** Comparison of PFS between patients with AFP<400 ng/ml and AFP≥400 ng/ml before the apatinib therapy. The mPFS was 8.7 months (95% CI: 6.95–10.46) for patients with AFP<400 ng/ml versus 5.0 months (95% CI: 4.44–5.56) for patients with AFP≥400 ng/ml (*p* < 0.05). **(C)** The mPFS was significantly longer in first-line (8.2 months, 95% CI: 7.39–9.01) and second-line (7.0 months, 95% CI: 5.27–8.73) treatment patients than in third-line (3.1 months, 95% CI: 1.71–4.50) treatment (*p* < 0.001). **(D)** The mPFS of apatinib combined with TACE (11.0 months, 95% CI: 8.32–13.68) and immunotherapy (8.0 months, 95% CI: 6.53–9.47) was longer than that of apatinib monotherapy (4.4 months, 95% CI: 3.63–5.17) (*p* < 0.001).**(E)** The mPFS was shorter in patients with distant (5.8 months, 95% CI: 4.50–7.10) and intrahepatic metastases (7.3 months, 95% CI: 4.97–9.63) than in patients with postoperative recurrence (11.3 months, 95% CI: 6.50–16.10) (*p* < 0.05), compared with different states of tumor progression.

**FIGURE 3:**
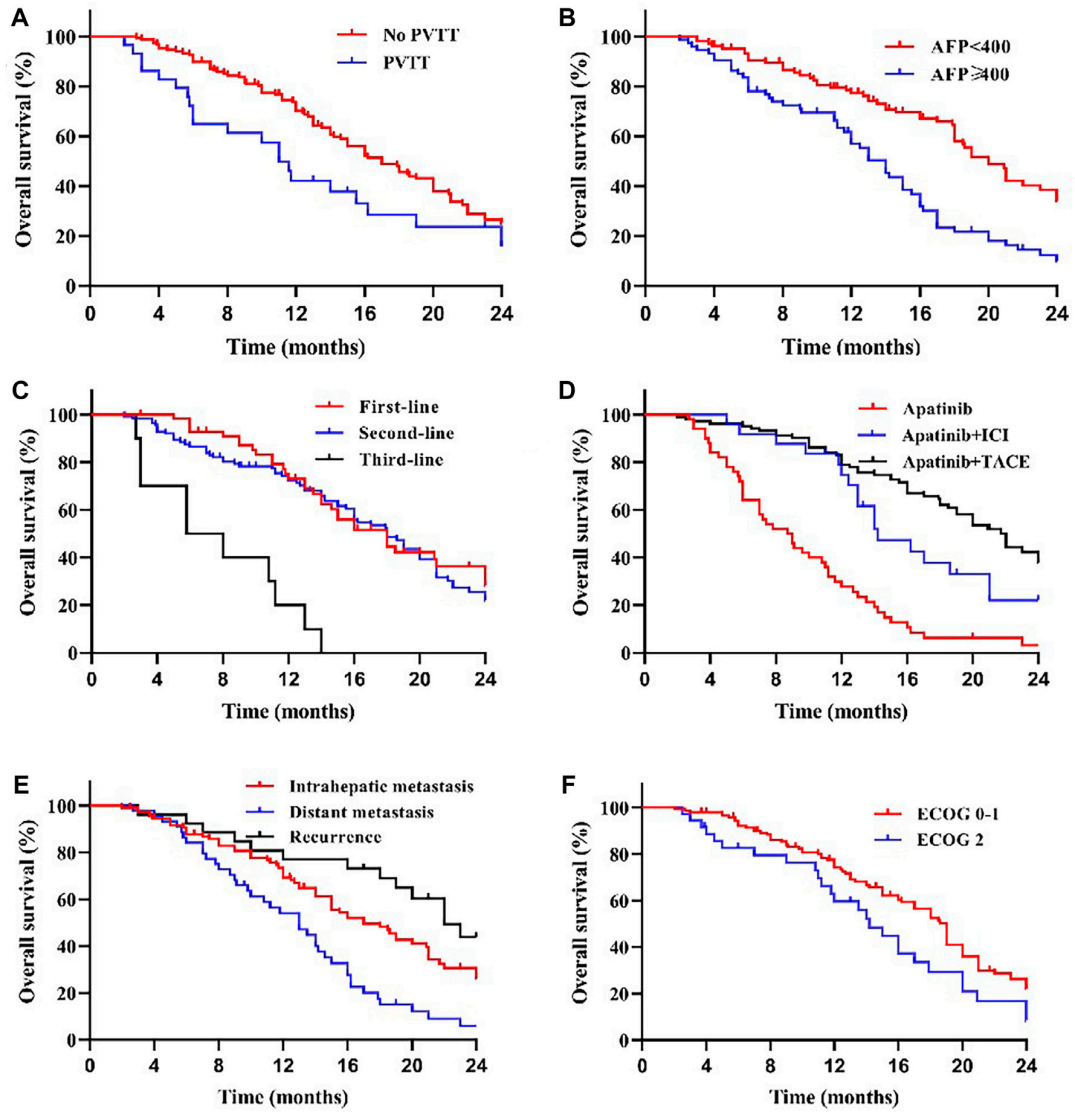
Kaplan–Meier curves showing the OS of patients by univariate analysis with advanced HCC treated with apatinib, including significantly different results. **(A)** Comparison of OS between patients with PVTT (present) and without PVTT (absent) before the apatinib therapy. The mOS was 11.0 months (95% CI: 8.90–13.10) for treatment with PVTT versus 16.0 months (95% CI: 13.42–18.58) for treatment without PVTT (*p* < 0.05). **(B)** Comparison of OS between patients with AFP<400 ng/ml and AFP≥400 ng/ml before the apatinib therapy. The mOS was 19.0 months (95% CI: 16.74–21.26) for patients with AFP<400 ng/ml versus 12.0 months (95% CI: 10.72–13.28) for patients with AFP≥400 ng/ml (*p* < 0.001). **(C)** The mOS was significantly longer in first-line (16.0 months, 95% CI: 12.61–19.39) and second-line (17.0 months, 95% CI: 14.33–19.67) treatment patients than in third-line (5.8 months, 95% CI: 0.64–10.97) treatment (*p* < 0.001). **(D)** The mOS of apatinib combined with TACE (20.0 months, 95% CI: 17.13–22.88) and immunotherapy (14.0 months, 95% CI: 12.58–15.42) was longer than that of apatinib monotherapy (8.7 months, 95% CI: 6.51–10.90) (*p* < 0.001). **(E)** The mOS was shorter in patients with distant (13.0 months, 95% CI: 10.09–15.91) and intrahepatic metastases (16.0 months, 95% CI: 12.54–19.46) than in patients with postoperative recurrence (24.0 months, 95% CI: 18.54–29.46) (*p* < 0.05), compared with different states of tumor progression. **(F)** Comparison of OS between patients with ECOG scores 0–1 and 2 before the apatinib therapy. The mOS was 16.2 months (95% CI: 13.33–19.07) for patients with ECOG scores 0–1 versus 13.5 months (95% CI: 10.39–16.61) for patients with ECOG scores 2 (*p* < 0.05).

**FIGURE 4:**
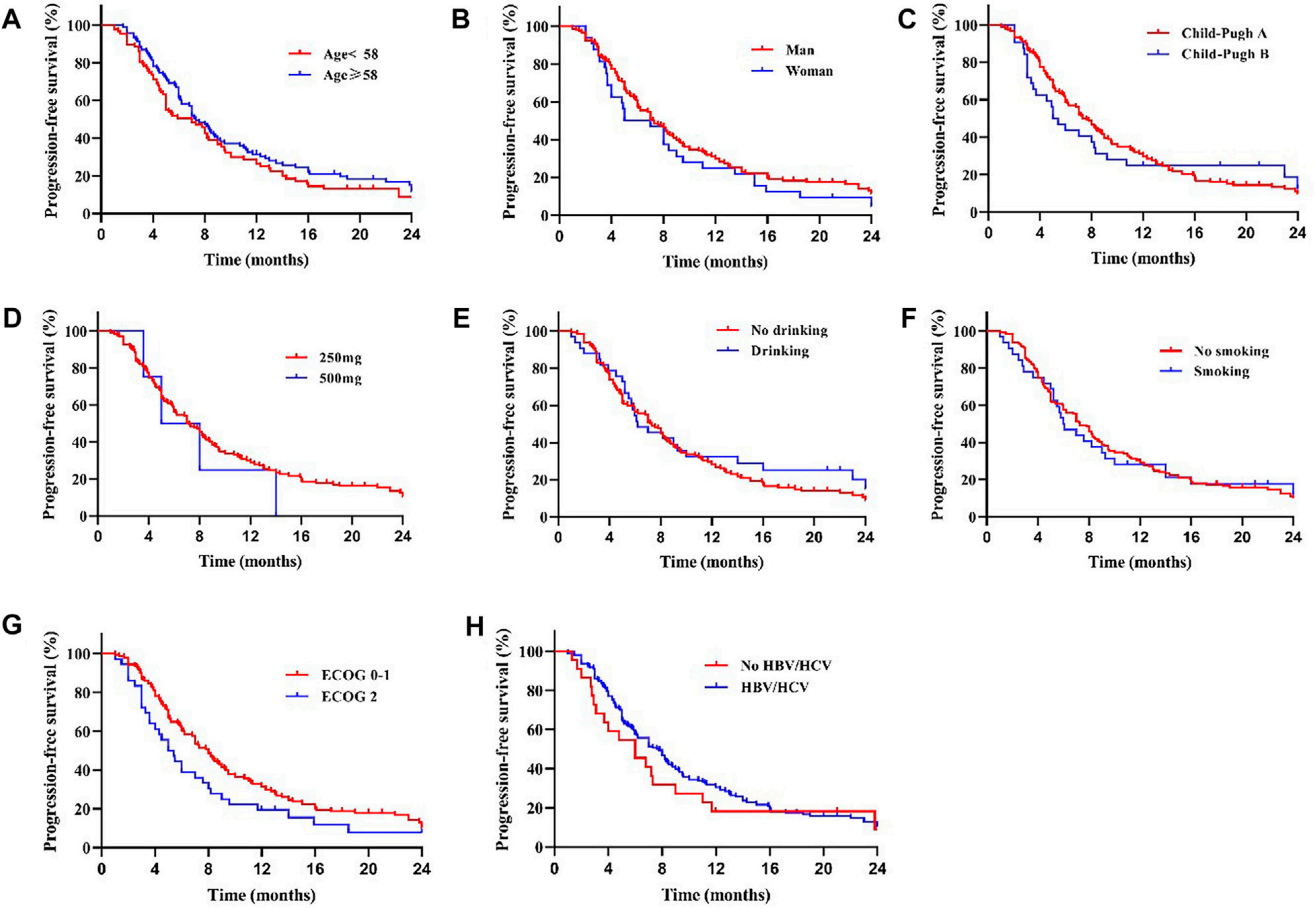
There was no significant difference in mPFS between patients with different age **(A)**, sex **(B)**, Child-Pugh class **(C)**, drug dose **(D)**, whether with drinking **(E)** or smoking **(F)** history, ECOG scores **(G)** and whether with HBV/HCV **(H)** (All *p* > 0.05).

**FIGURE 5:**
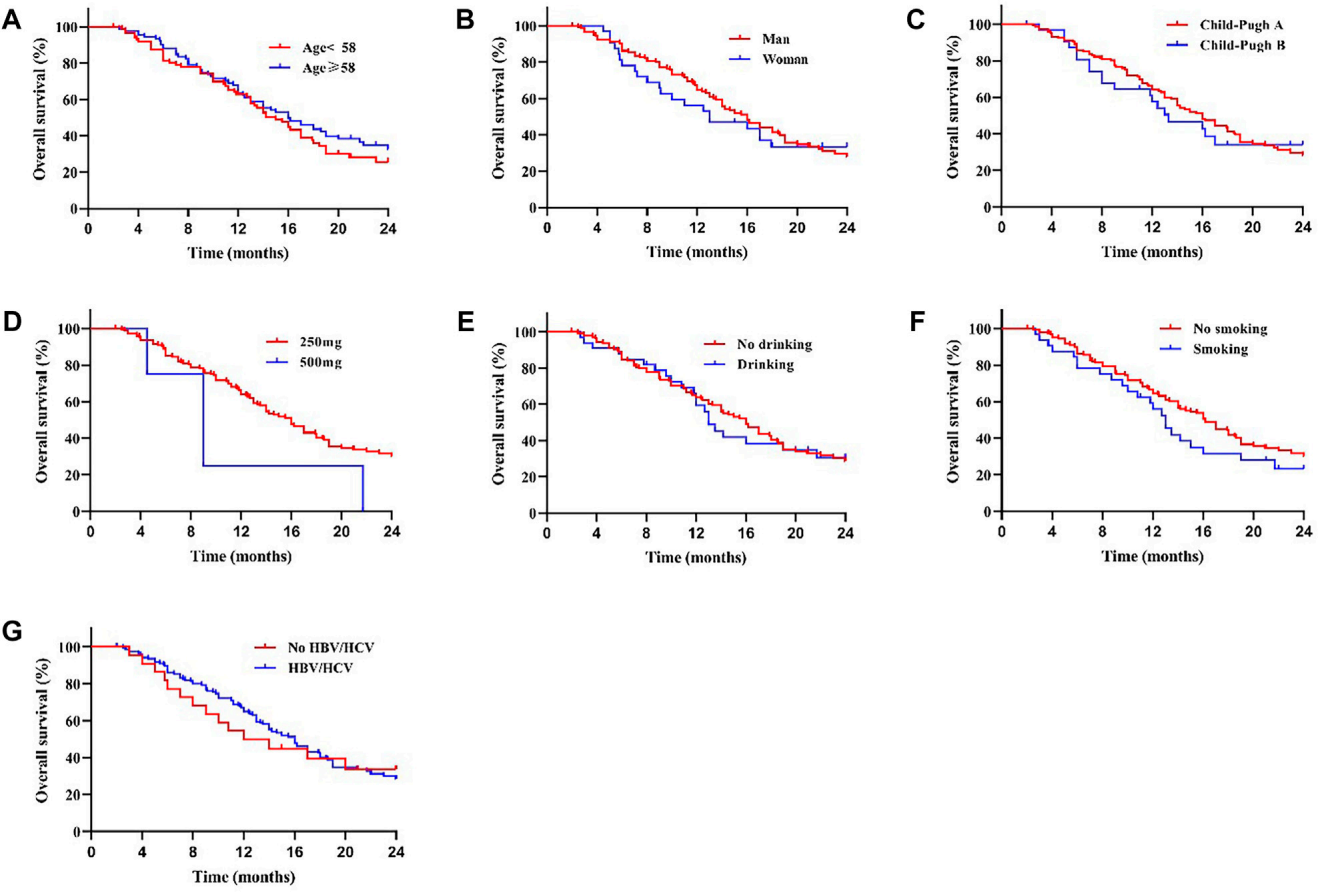
There was no significant difference in mOS between patients with different age **(A)**, sex **(B)**, Child-Pugh class **(C)**, drug dose **(D)**, whether with drinking **(E)** or smoking **(F)** history, and whether with HBV/HCV **(G)** (All *p* > 0.05).

We compared the survival outcomes of the different prognostic factors. Univariate analysis showed that line of apatinib (The lines of apatinib treatment), AFP, tumor progression, portal vein tumor thrombus (PVTT), and combination therapy may impact the PFS of patients. The patients without PVTT (8.0 months) showed longer mPFS compared with patients with PVTT (4.8 months) (*p* < 0.05) (Figure 2A). AFP level was an important factor affecting the prognosis of HCC patients. In our study, the mPFS was 8.7 months for patients with AFP<400 ng/ml and 5.0 months for patients with AFP≥400 ng/ml (*p* < 0.05) (Figure 2B). In addition, the mPFS was significantly longer in first-line (8.2 months) and second-line (7.0 months) treatment patients than third-line (3.1 months) (*p* < 0.001) (Figure 2C). Compared with apatinib monotherapy (4.4 months), the mPFS of combined therapy (combined with TACE and immunotherapy) (11.0 and 8.0 months) were longer (*p* < 0.001) (Figure 2D). Morever the mPFS was shorter in patients with distant (5.8 months) and intrahepatic metastases (7.3 months) than recurrence (11.3 months) (*p* < 0.05) (Figure 2E). In general, patients with third-line treatment, AFP ≥400 ng/ml, distant metastasis, PVTT, or apatinib monotherapy had shorter survival. For OS, univariate analysis showed that ECOG scores, line of apatinib, AFP, tumor progression, PVTT, and combination therapy may have impacts on it. Compared the patients with PVTT (11.0 months) with that without PVTT (16.0 months), there was significant difference in mOS between them (*p* < 0.05) (Figure 3A). And the mOS was 19.0 months for patients with AFP<400 ng/ml versus 12.0 months for patients with AFP≥400 ng/ml (*p* < 0.001) (Figure 3B). As the results showed, mOS was significantly longer in first-line (16.0 months) and second-line (17.0 months) treatment patients than in third-line (5.8 months) treatment (*p* < 0.001) (Figure 3C). Furthermore, the mOS of apatinib combined with TACE (20.0 months) and immunotherapy (14.0 months) were significant longer than that of apatinib monotherapy (8.7 months) (*p* < 0.001) (Figure 3D). The mOS also showed difference in different states of tumor progression. The mOS was shorter in patients with distant (13.0 months) and intrahepatic metastases (16.0 months) than in patients with postoperative recurrence (24.0 months) (*p* < 0.05) (Figure 3E). Unlike mPFS, the mOS was 16.2 months for patients with ECOG scores 0–1 versus 13.5 months for patients with ECOG scores 2 with significant difference (*p* < 0.05) (Figure 3F). However, according to our results, there were no significant differences in the mPFS and mOS of patients at different ages, sex, drug dose, Child–Pugh class, smoking or drinking history, and HBV/HCV status. The mPFS of patients with different ages (<58 vs. ≥58) were 7.0 and 7.3 months (*p* = 0.204) (Figure 4A), and the mOS were 15.0 and 16.0 months (*p* = 0.384) (Figure 5A), respectively. The mPFS of patients with different sexes (man vs. woman) were 7.2 and 5.0 months (*p* = 0.271) (Figure 4B), and the mOS were 16.0 and 13.0 months (*p* = 0.428) (Figure 5B), respectively. The mPFS and mOS of patients with Child-Pugh A were 7.3 and 16.0 months, and that with Child-Pugh B were 5.0 and 13.3 months (*p* = 0.787 and *p* = 0.809) (Figures 4C, 5C). The mPFS and mOS did not show statistical differences, possibly due to the limited number of patients taking 500 mg apatinib. The mPFS of patients with different apatinib doses (250 vs. 500 mg) were 7.0 and 5.0 months (*p* = 0.509), and the mOS were 16.0 and 9.0 months (*p* = 0.08) (Figures 4D, 5D). The mPFS was 7.3 months for patients with drinking history and 6.2 months for patients without drinking history (*p* = 0.501) (Figure 4E). The mOS was 16.0 months for patients with drinking history and 13 months for patients without drinking history (*p* = 0.883) (Figure 5E). The mPFS and mOS was 7.3 and 16.2 months for patients with smoking history, and 6.0 and 13.0 months for patients without smoking history, without statistical difference (*p* = 0.75 and *p* = 0.316) (Figures 4F, 5F). Patients with ECOG score 0–1 had a longer mPFS than those with ECOG score 2 (8.0 vs. 5.0 months), but the results were not statistically significant (*p* = 0.063) (Figure 4G). In addition, the mPFS was 6.0 months for patients with HBV/HCV and 7.6 months for patients without HBV/HCV (*p* = 0.335) (Figure 4H). And the mOS was 12.0 months for patients with HBV/HCV and 16.0 months for patients without HBV/HCV (*p* = 0.647) (Figure 5G).

Multivariate analysis confirmed that third-line drugs (HR = 3.21; 95% confidence interval (CI), 1.54–6.68; *p* < 0.05), PVTT (HR = 1.75; 95% CI, 1.13–2.70; *p* < 0.05), and combination therapy (Apatinib (HR = 3.70; 95% CI, 1.13–2.70; *p* < 0.001), particularly Apatinib + immunotherapy (HR = 1.91; 95% CI, 1.19–3.06; *p* < 0.05)), were independent prognostic factors for PFS in all patients (Table 3). And multivariate analysis proved AFP (HR = 1.61; 95% CI, 1.11–2.33; *p* < 0.05), with PVTT (HR = 1.70; 95% CI, 1.05–2.74; *p* < 0.05), and combination therapy (Apatinib (HR = 4.34; 95% CI, 2.88–6.53; *p* < 0.001)) were independent prognostic factors of OS (Table 4).

**TABLE 3:** Multivariable Cox proportional hazard models of progression.

Covariate	HR (95%Cl)	*p* value
Line of apatinib	–	–
First-line	–	–
Second-line	1.19 (0.84,1.69)	0.327
Third-line	3.21 (1.54,6.68)	0.002
PVTT	–	–
Absent	–	–
Present	1.75 (1.13,2.70)	0.011
Combination therapy	–	–
Apatinib	3.70 (2.48,5.52)	<0.001
Apatinib + immunotherapy	1.91 (1.19,3.06)	0.008
Apatinib + TACE	–	–

**TABLE 4:** Multivariable Cox proportional hazard models of mortality.

Covariate	HR (95%Cl)	*p* value
AFP	LR	–
<400 ng/ml	–	–
≥400 ng/ml	1.61 (1.11,2.33)	0.012
PVTT	–	
Absent	–	–
Present	1.70 (1.05,2.74)	0.032
Combination therapy	–	–
Apatinib	4.34 (2.88,6.53)	<0.001
Apatinib + immunotherapy	1.62 (0.94,2.81)	0.085
Apatinib + TACE	–	–

### Safety of apatinib in the treatment of HCC

Safety analysis was performed on all patients (Table 5). The most common adverse reactions were secondary hypertension (29.21%), symptoms of fatigue (16.85%), hand and foot syndrome (16.29%), vomiting (14.04%), liver dysfunction (6.18%), and proteinuria (6.74%). Of the 178 patients, 2 patients were admitted for severe diarrhea, dehydration, or bradycardia, and 1 patient was admitted for severe bone marrow suppression, which led to treatment discontinuation. The other patients had grade 1 or 2 adverse events.

**TABLE 5:** All adverse reactions in the patients after taking apatinib.

Grade, n (%)					
Adverse events	Grades 1 (%)	Grades 2 (%)	Grades 3 (%)	Grades 4 (%)	Grades 5 (%)
Vomiting	12 (6.74%)	13 (7.30%)	0	0	0
Increased ALT/AST	5 (2.81%)	7 (3.93%)	0	0	0
Proteinuria	3 (1.69%)	8 (4.49%)	0	0	0
Fatigue	20 (11.24%)	10 (5.62%)	0	0	0
Hypertension	9 (5.06%)	43 (24.16%)	0	0	0
Decreased platelet	3 (1.69%)	6 (3.37%)	1 (0.56%)	0	0
Hand and foot syndrome	10 (5.62%)	19 (10.67%)	0	0	0
Diarrhea	4 (2.25%)	11 (6.18%)	1 (0.56%)	0	0
Hoarseness	3 (1.69%)	2 (1.12%)	0	0	0
Rash	5 (2.81%)	3 (1.69%)	0	0	0
Bradycardia	0	0	1 (0.56%)	0	0
Alopecia	2 (1.12%)	0	0	0	0
Headache	2 (1.12%)	0	0	0	0
Gastrointestinal hemorrhage	0	1 (0.56%)	0	0	0

ALT/AST, alanine aminotransferase/ aspartate aminotransferase; Hypertension, the hypertension here was defined as an increase in blood pressure compared to before apatinib.

## Discussion

HCC is the third leading cause of cancer death worldwide (1). Most patients with HCC are diagnosed with advanced cancer with intrahepatic or distant metastases. Multiple clinical trials have demonstrated the potential survival benefit of apatinib as first- or second-line treatment for patients with advanced HCC (Qin, 2014; Qin et al., 2021). Combinations of other antitumor therapies, including radiotherapy, immunotherapy, and TACE, can increase this survival benefit (28, 29). In this retrospective study, we investigated the safety and efficacy of apatinib in patients with advanced HCC in the real world. The mPFS and mOS of the 178 patients were 7.0 and 16.0 months, respectively. The mOS in this study was significantly higher than that of sorafenib in patients with refractory advanced HCC, as reported in previous studies (Llovet et al., 2008; Cheng et al., 2009; Bruix et al., 2012). The IMbrave150 trial showed that the mPFS in the atezolizumab plus bevacizumab group was 6.8 months, and in the sorafenib group, it was 4.3 months (Finn et al., 2020). In our study, the mPFS of apatinib monotherapy was 4.4 months, close to that of sorafenib. In the case of combined immunotherapy, mPFS reached 8.0 months, even more than atezolizumab plus bevacizumab. The mPFS was 5.7 and 5.5 months for first- and second-line treatment of unresectable HCC with apatinib in combination with camrelizumab in the RESCUE trial (29). In our study, the immune checkpoint inhibitors in immunotherapy combinated apatinib included not only camrelizumab but also other drugs such as durvalumab, which may be responsible for the longer mPFS. In the RESORCE trial, regorafenib provided an OS of 10.6 months for patients with sorafenib progression, compared with a mOS of 8.7 months for apatinib monotherapy in our study, which was close to the efficacy of regorafenib monotherapy (Bruix et al., 2017b). The overall mPFS and mOS were significantly longer than those found in other retrospective studies involving apatinib (mPFS: 7.0 vs. 5.0 months; mOS: 16.0 vs. 13.0 months), especially as a second-line treatment; this may be due to the combination with immunotherapy (mOS: 14.0 months; mPFS: 8.0 months) or TACE (mOS: 20.0 months; mPFS: 11.0 months) in some patients in this study (Zhen et al., 2018; Qin et al., 2021). In a recent single-center retrospective study about unresectable HCC, apatinib plus camrelizumab demonstrated the mOS of 13.1 months, which was similar to our results (14.0 months) (Ju et al., 2021). Similar to other studies, apatinib combined with TACE significantly improved mPFS and mOS. In a study of combination of TACE and apatinib for the treatment of HCC,the mOS and mPFS in the combination group were only 10.0 and 5.5 months, lower than the 20.0 and 11.0 months in our study (Liu et al., 2021). In their study, most HCC was accompanied by vascular invasion or distant metastasis, which were the important factors impairing the prognosis of patients (Yang et al., 2019b). The promising efficacy of apatinib may be due to its ability to selectively target VEGFR-2 and a higher binding affinity than sorafenib. TACE induces hypoxia in HCC tissues and increases the level of VEGF in the remaining HCC tissues, leading to a significant neovascularization response (Tian et al., 2011). Therefore, the combination of apatinib and TACE can improve the efficacy.

In univariate analysis, there were no significant differences in mPFS and mOS between patients with different ages, sex, drug dose, Child–Pugh class, smoking or drinking history, and HBV/HCV status. Patients with ECOG score 0–1 had a longer mPFS than those with ECOG score 2, but the results were not statistically significant; meanwhile, different ECOG scores had a significant impact on mOS. HBV/HCV infection and drinking showed no influence on patients’ survival, which may be because viral infection and alcohol consumption are the pathogenic factors of HCC, and antiviral therapy and alcohol abstention are carried out after the diagnosis of HCC (Chong et al., 2015). The effect of HBV/HCV on the prognosis of HCC patients may be related to the treatment mode, and the current research is controversial (Papatheodoridi et al., 2021).It has been reported that in a population of HCC patients undergoing surgical resection, the presence or absence of HBV/HCV infection has no effect on patient prognosis (Pawlik et al., 2004; Li et al., 2013; Nishikawa et al., 2013). This result was interpreted as tumors in patients with HBV/HCV infection were more likely to form an envelope, limiting tumor growth (Pawlik et al., 2004; Li et al., 2013; Nishikawa et al., 2013). Previous studies have also shown that the presence of HBV/HCV in patients with advanced HCC does not adversely affect the efficacy of apatinib or sorafenib, but may even be beneficial to it (3). 60–90% of HCCs develop in persons with basic liver disease, which may lead persons to stop consuming alcohol and smoking, and may bias the true association (Turati et al., 2014; Petrick et al., 2018). Since only 4 patients received the 500 mg dose, with no significant survival difference, they were excluded from the multivariate analysis to avoid bias. In this retrospective study, AFP, PVTT, and combination therapy were identified as independent prognostic factors for OS, while line of apatinib, PVTT, and combination therapy were independent prognostic factors for PFS.

Among all patients, mPFS was significantly longer in first-line and second-line treatment patients than in third-line treatment patients, suggesting that earlier application of apatinib for HCC may lead to better survival (Qin, 2014). In the 28 patients with PR, 27 had 250 mg of apatinib as their first/second-line treatment. Early application of apatinib had better antitumor effect. However, there was no difference in survival between first-line and second-line treatment groups; this may be related to the choice of combination therapy. Unfortunately, although univariate analysis showed that different line of apatinib had an effect on the OS of patients with advanced HCC, Cox regression analysis did not show the same results. Extrahepatic metastasis is a known risk factor for patients with HCC, and this was supported by the results of the univariate analysis. AFP level and PVTT were considered important factors affecting the survival and prognosis of patients with HCC (Tandon and Garcia-Tsao, 2009). In our study, patients with AFP ≥400 ng/ml had 1.61-fold the risk of death compared with patients with AFP <400 ng/ml, while patients with PVTT had a 1.75-fold and 1.70-fold risk of progression and death, respectively, compared with patients without PVTT. In contrast, survival and prognosis were significantly worse in patients with venous PVTT, which is consistent with previous studies (3, 49).

Our study confirmed that apatinib combined with immunotherapy or TACE can significantly improve efficacy. In the 28 patients with PR, 21 patients were treated with apatinib combined with immunotherapy or TACE. Especially for patients with PVTT, apatinib combined with TACE has a longer survival than TACE alone (Fan et al., 2019). Numerous clinical studies have corroborated the benefits of TACE plus apatinib for advanced HCC. TACE therapy can create an anoxic environment for the tumor, which induces high VEGFR expression and angiogenesis; this is why the combination with apatinib is so effective (Pinato et al., 2018). Recently, the RESCUE trial showed that apatinib therapy combined with camrelizumab was controllable, safe, and showed a good curative effect in advanced HCC (Xu et al., 2021). The ORR was 34.3% in the first-line and 22.5% in the second-line cohort. mPFS in the cohorts were 5.7 and 5.5 months, respectively. Meanwhile, the mPFS of patients with HCC treated with combination immunotherapy was 8.0 months, which may be due to the use of multiple immunotherapy drugs, including carrelizumab and attilizumab, in our enrolled patients.

Apatinib, similar to other molecularly targeted drugs, can cause adverse reactions; this was a focus of the current study as well. Detailed information regarding this is shown in Table 5 The most common adverse reactions were secondary hypertension (29.21%), symptoms of fatigue (16.85%), hand and foot syndrome (16.29%), vomiting (14.04%), liver dysfunction (6.18%), and proteinuria (6.74%). These adverse reactions could be tolerated or were alleviated with symptomatic treatment. Of the 178 patients, 2 patients were admitted for severe diarrhea, dehydration, proteinuria, or bradycardia, and 1 patient was admitted for severe bone marrow suppression, which led to treatment discontinuation. The other patients experienced grade 1 or 2 adverse events. Hypertension, proteinuria, and reduced liver function were more common in the apatinib treatment group, while sorafenib was more likely to cause hand-foot syndrome (Zhao et al., 2016). The adverse effects of apatinib are relatively mild compared to other cytotoxic chemotherapies and targeted therapies. No deaths associated with apatinib treatment or irreversible organ or tissue damage were observed in our study. However, possible adverse reactions associated with apatinib still require further study.

This study had some limitations. First, this was a retrospective pilot study with patients from Jilin, Liaoning, Heilongjiang, and Inner Mongolia provinces. Second, the doses of apatinib taken by patients were mostly 250 mg, which was determined by the physician based on weight, general condition, and tolerability; it was significantly lower than the doses used in previous studies on gastric and breast cancer. Only 4 patients in this study received the 500 mg dose, therefore, it was difficult to elucidate the differences between the effects at various doses. Third, both HBV and HCV are important pathogenic factors of HCC, however, we did not analyze the two factors separately. Fourth, this is a single-arm study with a small sample size, and large randomized controlled trials are still needed to explore the efficacy and safety of apatinib for advanced HCC.

## Conclusion

The result of our study suggested that apatinib is effective in treating advanced HCC. Apatinib combined with TACE or immunotherapy increased the survival benefit for patients with advanced HCC. The most common adverse reactions occurred in patients were secondary hypertension, gastrointestinal resistance, symptoms of fatigue, and hand and foot syndrome, which were tolerable and manageable.

## Data Availability

All data generated or analyzed during this study are included in this published article. The datasets used or analyzed during the current study are available from the corresponding author on reasonable request.

## Abbreviations

HCC, hepatocellular carcinoma; HBV/HCV, hepatitis B/C virus; AFP, alpha fetoprotein; PVTT, portal vein tumor thrombus; TACE, transcatheter arterial chemoembolization; VEGF, vascular endothelial growth factor; TKI, tyrosine kinase inhibitor; FDA, Food and Drug Administration; VEGFR-2, vascular endothelial growth factor receptor 2; RECIST, Response Evaluation Criteria in Solid Tumors; CTCAE, Common Terminology Criteria for Adverse Events; ECOG, Eastern Cooperative Oncology Group; PFS, progression-free surviva; lOS, overall survival; CR, complete response; PR, partial response; SD, stable disease; PD, Progressive disease; DCR, disease control rate; ORR, overall response rate; CI, confidence interval; ALT/AST, alanine aminotransferase/aspartate aminotransferase.
