# Supplementary figures and images for: Efficacy and Safety of Apatinib in Advanced Hepatocellular Carcinoma: A Multicenter Real World Retrospective Study
Source: Front Pharmacol. 2022 May 17;13:894016. doi: 10.3389/fphar.2022.894016 (PMC9152289; doi:10.3389/fphar.2022.894016)

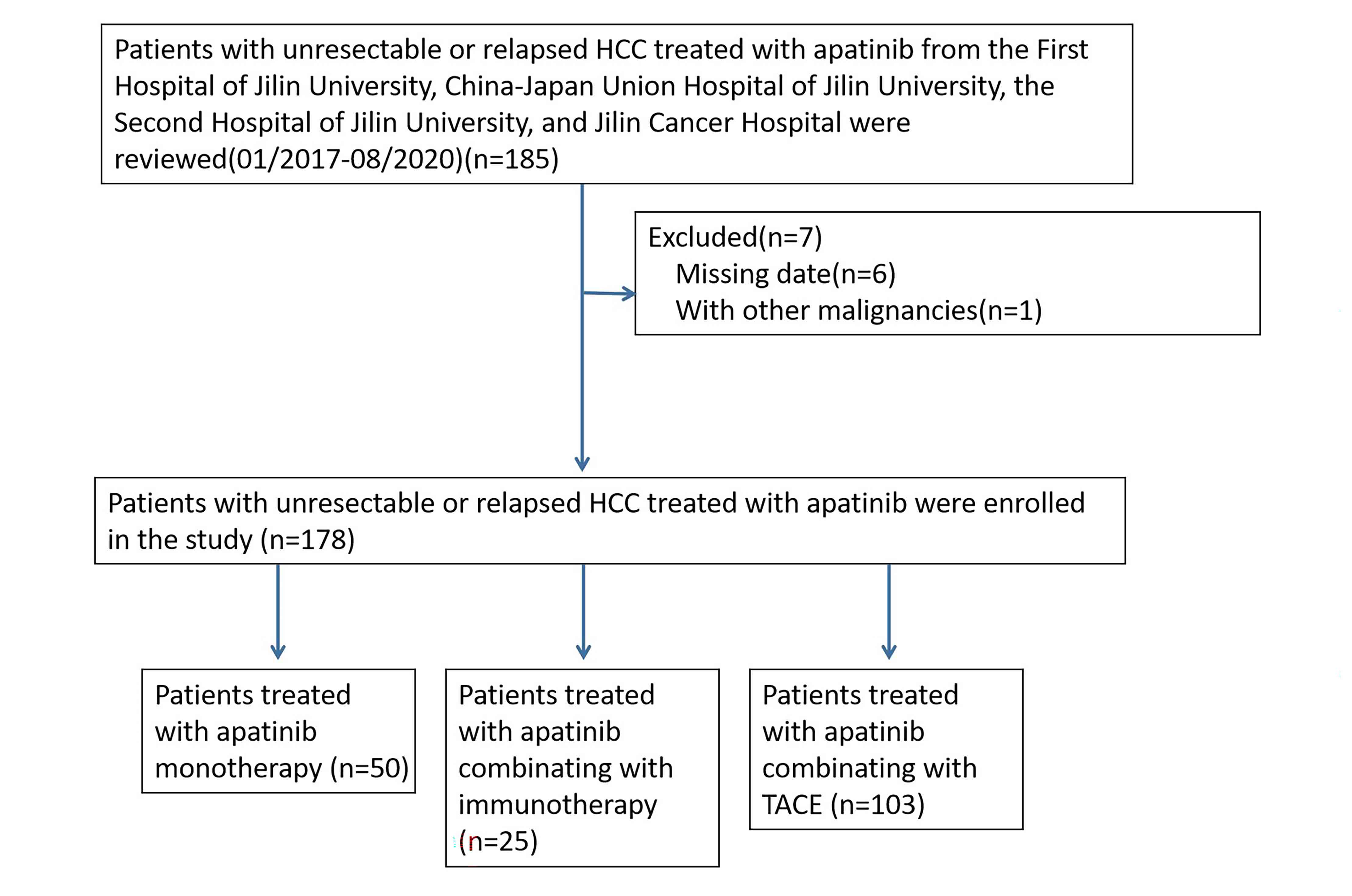

Supplement: Supplementary file 1 [file Image1.JPEG]
